# The gastrin-releasing peptide receptor as a potential biomarker for squamous cell carcinoma

**DOI:** 10.1038/s41598-025-24861-4

**Published:** 2025-11-20

**Authors:** Danielly Brufatto Olguins, Júlia Caroline Marcolin, Mariane da Cunha Jaeger, Manoela Domingues Martins, Luis Fernando da Rosa Rivero, Gilberto Schwartsmann, Rafael Roesler, Marcelo Gerardin Poirot Land, Martina Lichtenfels, Caroline Brunetto de Farias

**Affiliations:** 1 Cancer and Neurobiology Laboratory, Experimental Research Center, Clinical Hospital (CPE-HCPA), Porto Alegre, RS Brazil; 2 Graduate Program in Biological Sciences: Pharmacology and Therapeutics, Federal University of Rio Grande do Sul, Porto Alegre, RS Brazil; 3 Children’s Cancer Institute, Porto Alegre, RS Brazil; 4 School of Dentistry, Federal University of Rio Grande do Sul, Porto Alegre, RS Brazil; 5 Department of Pathology, School of Medicine, Federal University of Rio Grande do Sul, Porto Alegre, RS Brazil; 6 Department of Internal Medicine, School of Medicine, Federal University of Rio Grande do Sul, Porto Alegre, RS Brazil; 7 Department of Pharmacology, Institute for Basic Health Sciences, Federal University of Rio Grande do Sul, Porto Alegre, RS Brazil; 8 Post-Graduate Program in Maternal and Child Health, Federal University of Federal University of Rio de Janeiro, Rio de Janeiro, Brazil; 9 National Science and Technology Institute for Children’s Cancer Biology and Pediatric Oncology – INCT BioOncoPed, Porto Alegre, Brazil; 10 Department of Translational Research, Ziel Biosciences, Porto Alegre, RS Brazil

**Keywords:** Gastrin-releasing peptide receptor, Squamous cell carcinoma, Head and neck neoplasms, Esophageal cancer, Cancer screening, Head and neck cancer, Tumour biomarkers

## Abstract

The gastrin-releasing peptide receptor (GRPR) is expressed across multiple human cancer types, including head and neck tumors, and has also been detected in the oral mucosa of patients bearing head and neck neoplasms. This study aimed to evaluate GRPR expression in tumor samples obtained from patients with histopathologically confirmed squamous cell carcinomas of the head and neck and esophagus. GRPR protein expression was analyzed by immunohistochemistry in 80 tumor specimens and 10 cancer-free tissue samples. GRPR positivity was observed in 72.5% of tumors, whereas all healthy control samples were negative. Additionally, an expression threshold of 10% was sufficient to distinguish between positive and negative tumors (*P* < 0.0001). Overall survival for the cohort was 64.7%. GRPR expression tends to influence patient outcomes, with an expression level exceeding 50% correlating with worse survival (*p* = 0.031). GRPR is overexpressed in head and neck and esophageal squamous cell carcinoma, and higher expression was associated with worse survival, suggesting a promising role of GRPR as a new biomarker for early diagnosis and prognosis.

## Introduction

Head and neck cancer, which includes malignancies of the oral cavity, pharynx, larynx and thyroid, is predicted to reach 1.98 million cases worldwide by 2030^1,2^. In Brazil, the National Cancer Institute (INCA) estimated 39,550 new head and neck cancer cases in 2023, including tumors of the mouth (oral cavity), larynx and thyroid^3^. Esophageal cancer is the seventh most common cause of cancer-related deaths worldwide, with incidence varying across countries and populations. This variation is associated with differences in risk-factor prevalence and histologic subtype distribution^4^. These tumors originate in squamous cells, which line moist surfaces^5^.

Several factors have been identified as risk factors for the development of squamous cell carcinomas (SCC), and they appear to act synergistically, primarily by increasing mutational burden. Exogenous factors related to lifestyle, especially tobacco and alcohol, are particularly significant. Additionally, in some cases, exposure to ultraviolet radiation, betel nut use, and human papillomavirus (HPV) infection have been described as associated factors^6,7^. Intrinsic contributors such as malnutrition, comorbid systemic conditions, age, gender, and heritable/oncogenic variants also modulate risk^8^. Recently, oropharyngeal cancer has been correlated with HPV, a sexually transmitted virus, increasing the risk in young individuals not exposed to tobacco^9^.

Each tumor is associated with unique etiology, and the treatment is defined based on the disease stage and location^10^. Surgery is standard in early-stage head and neck cancer, while radiotherapy, chemotherapy, targeted therapy, and immunotherapy are integrated as indicated^11^. The potential use of biomarkers includes early detection, risk stratification, and treatment selection, leading to increased survival. However, no specific biomarkers are currently known for head and neck and esophageal cancer.

Gastrin-releasing peptide (GRP) is a neuroendocrine peptide that belongs to a bombesin-like peptide family found in mammals and has been associated with cancer development^12,13^. Its effects are mediated through the gastrin-releasing peptide receptor (GRPR), a G-protein coupled receptor implicated in oncogenesis^14^. GRPR overexpression has been reported in prostate, breast, cervical, lung, head and neck, and gastric cancers^12,15^. The interaction of GRPR on the surface of GRP-expressing cells forms an autocrine loop, promoting tumor growth^16^. Despite promising results linking GRPR with head and neck and esophageal tumors, few data are available on GRPR immunohistochemistry and its correlation with patient survival^15,17,18^.

We investigated GRPR protein expression by immunohistochemistry in SCC of head and neck and esophageal tumors and evaluated whether GRPR serves as a prognostic biomarker in these tumors.

## Materials and methods

### Study population

A retrospective study was conducted at the Hospital de Clínicas de Porto Alegre between 2007 and 2011. The study encompassed 80 cases of SCC involving head and neck and esophageal tumors. Clinical data, including age, gender, tumor location, treatment details, recurrence, and follow-up, was reviewed from medical charts and hospital records. The study was reviewed and approved by the Research Ethics Committee of the Hospital de Clínicas de Porto Alegre, under the identification number 54314316.0.0000.5327, which also waived the requirement for informed consent. All procedures were performed in accordance with the following relevant guidelines and regulations.

### Tissue microarray (TMA)

TMA construction was performed using a self-embedding TMA template. Liquid paraffin placed at 65 °C for five minutes to mold. A heated 3 mm demo punch was used. A diagram was used to position the blocks in the TMA and to include these inverted blocks. The first blocks were warmed at 65 °C for six minutes, punctured with demo punch, and placed in the TMA template. TMA templates were filled with liquid paraffin and left at 70 °C for 8 min until the paraffin melted. The grill was placed on top, and the temperature reduced to cool, turning the paraffin a solid block.

### Immunohistochemical technique (IHC)

The primary antibody used was a rabbit polyclonal antibody anti-GRPR (Ziel Biosciences, Brazil). After dewaxing, inactivating endogenous peroxidase activity and blocking cross-reaction with normal serum, 3 μm sections were incubated overnight at 4 °C with a diluted solution of the primary antibody (1:400) (1:50). Identification of the location of the primary antibody was obtained by subsequent application of secondary antibodies, and visualization of the reaction obtained with Liquid Dab (Dako, K3468) and thus slides were stained with Harris hematoxylin and differentiated into 2% ammoniacal water and dehydrated in absolute alcohol and placed in xylol for assembly of the resin blades of the Entellan type. Samples with less than 10% of cell staining were considered negative, 10% to 50% cell staining were considered mild to moderate positive, while those with 51% to 100% cell staining were classified as strongly positive.

### Statistical analysis

Categorical variables were described as percentages, while continuous variables were presented as means (SE) and medians (IQR1-3). The cutoff point for distinguishing positive and negative GRPR expression was determined through receiver operating characteristic (ROC) curve analysis combined with the Youden index. The sensibility, specificity, positive predictive value, negative predictive value, and the cut-off point accuracy were calculated as usual in the site MedCalc’s Diagnostic test evaluation calculator. Overall Survival (OS) was defined as the time from diagnosis to death or the last follow-up date. The considered event was the death of any cause. Survival curves were calculated by Kaplan–Meier method and compared using the log-rank test. All statistical analyses were performed using the Statistical Package for the Social Sciences (SPSS) software program, version 20.0, SPSS Inc., Chicago, IL, USA.

## Results

### Patients and samples characteristics

The focus of this study was to assess GRPR expression in head and neck tumors, which most commonly originate in the squamous cells lining the mucosal surfaces of these sites. To this end, we analyzed specimens from two typical anatomic locations where these tumors are commonly found: the larynx and oral cavity. To explore potential site-specific differences in GRPR expression, we also included esophageal squamous cell carcinoma. The clinicodemographic characteristics of patients with head and neck and esophageal SCC are detailed in Table 1. The average age of the cohort was 59 years, with a predominance of males (83.8%). Primary tumor sites were the esophagus (50%), larynx (33.8%), and oral cavity (16.3%). A significant portion of the patients were identified as current smokers (60%) or former smokers (30.8%), and alcohol use was documented in 47.5%.

**Table 1:** Clinico-demographic features of patients included in the present study (*n* = 80).

Characteristics	*N*	%
Age		
Mean (SE)	59 (1.3)	
Median (IQR_1-3_)	57 (15.0)	
Sex		
Male	67	83.8
Female	13	16.3
Tumor site		
Esophagus	40	50
Larynx	27	33.8
Oral cavity	13	16.3
Tobacco user^a^		
Yes	39	60
Former smoker	20	30.8
No	6	9.2
Alcohol user^b^		
No	26	40.6
Former drinker	19	29.7
Yes	19	29.7
Surgery^c^		
Yes	39	56.5
No	30	43.5
Chemotherapy^c^		
Yes	26	37.7
No	43	62.3
Radiotherapy^c^		
Yes	51	73.9
No	18	26.1
Events^c^		
Recurrence	10	28.6
Metastasis	14	40
Inoperable with progression	10	28.6
New primary	1	2.9
Inoperable tumor^d^		
Progression	10	58.8
Stable	7	41.2
Survival^e^		
Alive	47	66.2
Death	24	33.8

^a^15 missing; ^b^16 missing; ^c^11 missing; ^d^13 missing; ^e^9 missing.

Of the 69 patients with complete clinical data, 39 (56.5%) had operable tumors and underwent surgery, whereas 30 (43.5%) had inoperable tumors. Radiotherapy was the primary treatment choice (73.9%), and 37.7% received chemotherapy. Over a mean follow-up of 40 months, 10 patients developed disease recurrence, and 14 developed distant metastases. Additional clinical details are provided in Table 1.

### GRPR protein is more expressed in head and neck squamous cell carcinoma compared to normal oral mucosa

**Fig. 1:**
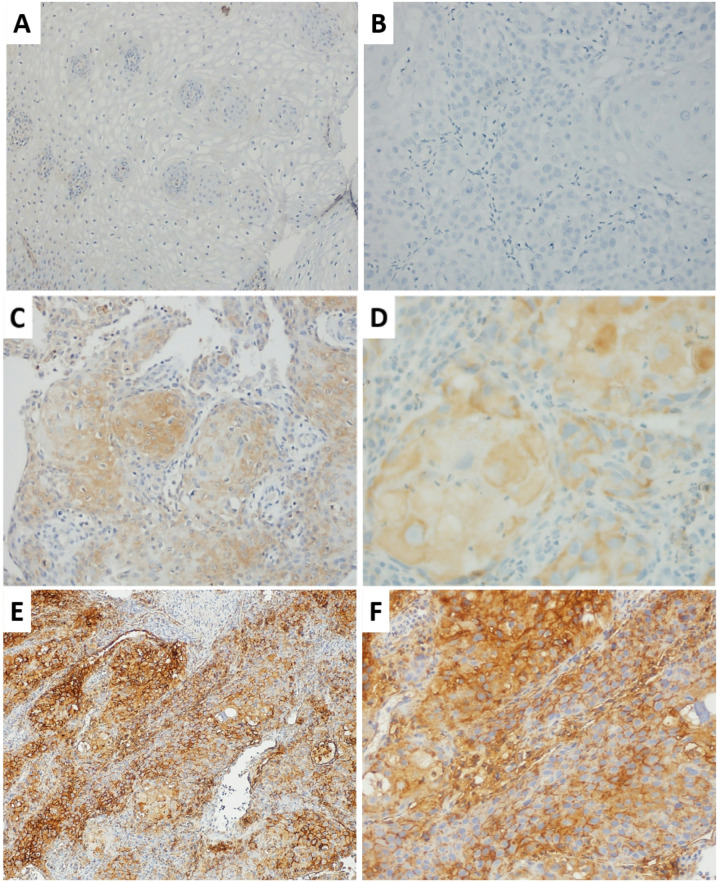
Immunohistochemical analysis of GRPR expression using a GRPR-specific antibody, showing representative staining patterns across varying magnifications. (**A**) Negative staining for GRPR is shown at a magnification of 100x and (**B**) 200x. (**C**) Mild to moderate cytoplasmic GRPR expression is observed at a magnification of 100x and (**D**) 400x. (**E**) Strong membrane and cytoplasmic GRPR staining is visible at a magnification of 100x and (**F**) 200x.

GRPR expression was evidenced by a diffuse and stronger staining in 67.2% of head and neck SCC samples compared to healthy tissue (*p* = 0.00003) (Fig. 1). Healthy tissues showed no GRPR expression. The anti-GRPR antibody showed 67% sensibility, 100% specificity and 70% accuracy to detect GRPR.

### GRPR potential as a marker for squamous cell carcinoma

GRPR expression in cancer and control samples was assessed by immunohistochemistry using an anti-GRPR antibody developed by Ziel Biosciences, Brazil. Our findings revealed that 58 (72.5%) cancer samples were GRPR-positive (GRPR+), and 22 (27.5%) were GRPR-negative (GRPR-). Notably, no healthy controls showed GRPR+ (Table 2). Additionally, the test yielded a sensitivity of 72.5%, specificity of 100%, and a positive predictive value of 100%, highlighting its potential for accurate screening in patients with positive GRPR expression. The cut-off point accuracy was 75.6%.

**Table 2:** GRPR expression in patient biopsy and control samples.

	Squamous cell carcinoma			
Test	Present	n	Absent	n
GRPR mild-moderate positive	True positive	41	False positive	0
GRPR strongly positive	True positive	17	False positive	0
GRPR negative	False negative	22	True negative	10
Total		80		10

**Fig. 2:**
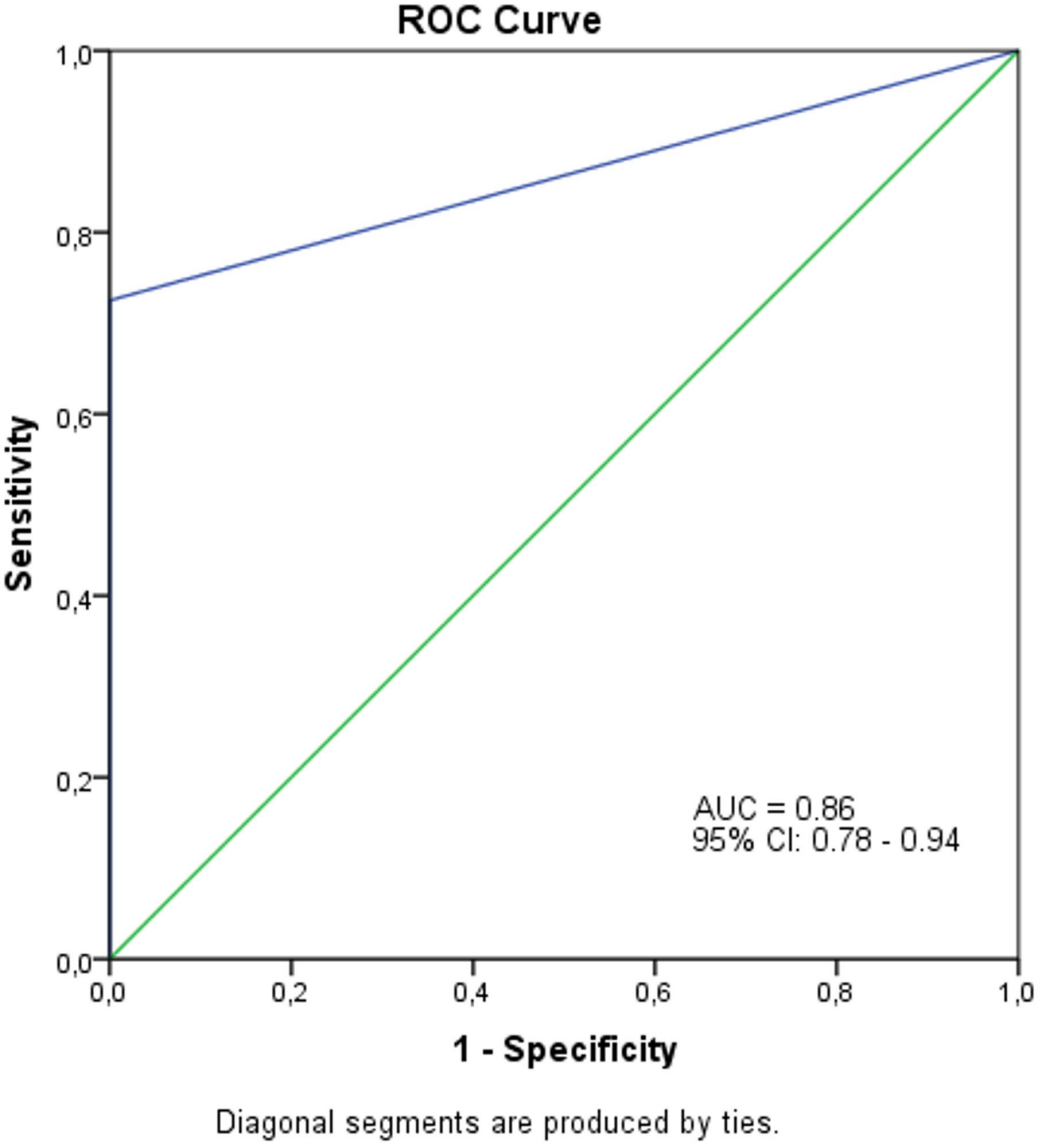
ROC curve illustrating the performance of GRPR expression in distinguishing squamous cell carcinoma cases from non-cancer cases. The area under the curve (AUC) is 0.86, with a 95% confidence interval of 0.78 to 0.94, indicating a strong accuracy of GRPR expression as a predictive marker for squamous cell carcinoma. ROC, receiver operating characteristic.

A GRPR expression level as low as 10% was sufficient to distinguish positive from negative tumor samples (*p* < 0.0001). This threshold was established through ROC curve analysis, which demonstrated an AUC of 0.86 (95% CI, 0.78–0.94; (Fig. 2), confirming good discriminative performance. Taken together, these findings support GRPR as a promising diagnostic biomarker for head and neck and esophageal SCC.

### GRPR expression is an indicator of worse survival

**Fig. 3:**
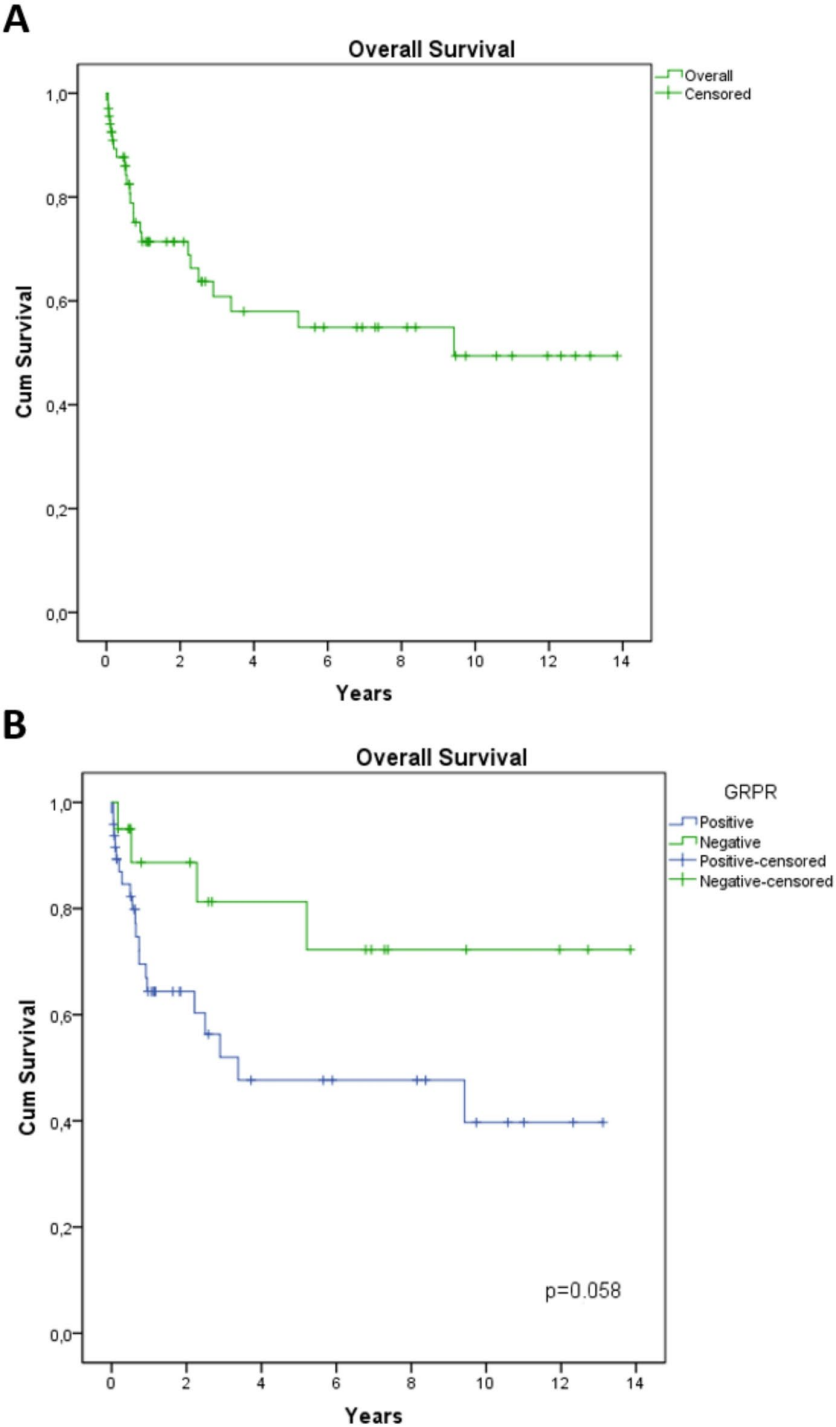

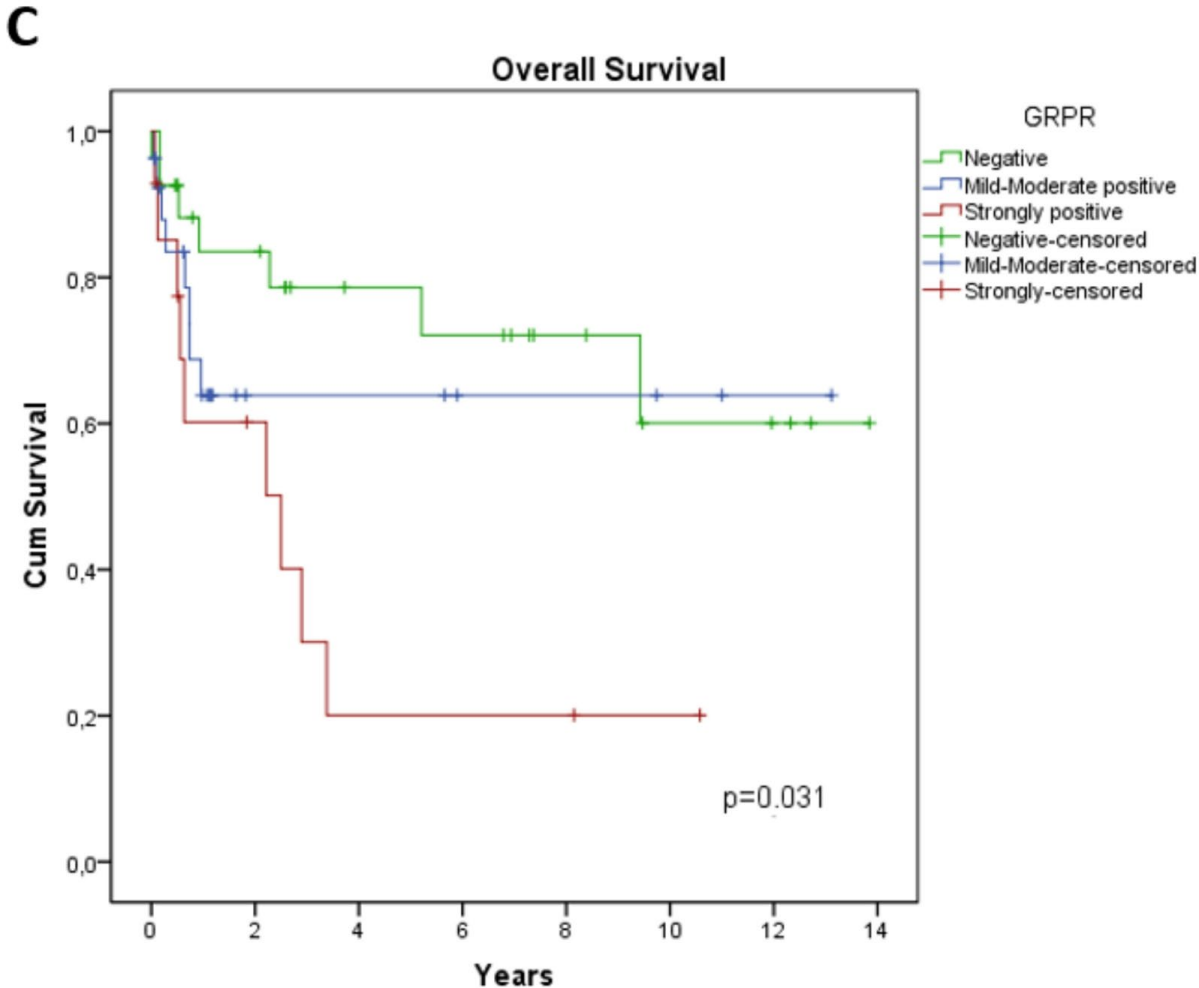
Overall survival curves of patients with head and neck, and esophageal squamous cell carcinoma, categorized by GRPR expression. (**A**) Kaplan-Meier curve representing the overall survival of all patients in the cohort. (**B**) Survival curves stratified by GRPR expression status, showing comparisons between GRPR-positive and GRPR-negative patients. (**C**) Overall survival curves categorized by levels of GRPR expression: negative, mild-to-moderate positive, and strongly positive expression.

The overall survival rate was 64.7% (Fig. 3A). When comparing GRPR + and GRPR- tumors, the difference in survival approached significance (*p* = 0.058) (Fig. 3B), potentially due to the limited sample size. However, when stratified by the percentage of stained cells, strongly positive GRPR expression was significantly associated with poorer survival (*p* = 0.031) (Fig. 3C). This finding suggests that high GRPR expression may influence outcomes and support its potential utility as a prognostic biomarker in this disease.

## Discussion

Gastrin-releasing peptide (GRP) exerts numerous physiological functions, including gastrointestinal hormone release, smooth muscle contraction, pancreatic enzyme secretion, and also acts as a neurotransmitter in the central nervous system. These effects are mediated by the gastrin-releasing peptide receptor (GRPR), a member of the G-protein receptor superfamily^14^. GRPR activity involves multiple signaling pathways, with phospholipase C (PLC) activation being one of the primary mechanisms, leading to intracellular calcium mobilization and protein kinase C (PKC) activation^19^. Additionally, GRPR signaling engages other intracellular mediators, including mitogen-activated protein kinases (MAPKs), adhesion kinases, phosphatidylinositol 3-kinases (PI3K), and cyclic AMP response element-binding proteins (CREB)^20,21^. Through these pathways, GRPR contributes to the regulation of cellular processes such as proliferation, survival, and migration.

GRP/GRPR signaling has been implicated in various cancers^22^. GRPR overexpression is reported in breast^23^, cervical^24^, anal canal^25^, and lung cancers^26^, whereas healthy tissues typically exhibit low or absent expression. Notably, GRPR immunostaining has demonstrated high sensitivity, specificity, and accuracy for detecting invasive cervical cancer, suggesting its value as a diagnostic biomarker^27^.

Consistent with previous findings, our research indicates GRPR overexpression in 72.5% of head and neck and esophageal SCC, with no expression in healthy controls. Lango^18^ and colleagues similarly observed elevated GRPR mRNA in tumors and adjacent normal mucosa from patients with head and neck SCC (HNSCC) compared with normal mucosa from cancer-free individuals (*P* < 0.001). In HNSCC, stimulation of GRPR by its ligand GRP promotes dose-dependent proliferation and MAPK activation, whereas disruption of the GRP-GRPR axis inhibits cell growth in vitro and in vivo, consistent with an autocrine regulatory pathway and suggesting that increased GRPR expression may be an early event in HNSCC development^18,28^.

Similarly, Fang^17^ and colleagues found mRNA GRP overexpression in 10 out of the 12 (83.3%) esophageal SCC samples compared to adjacent normal samples. More recently, GRPR was successfully targeted using a nanoparticle-based delivery system in oral carcinoma cell lines to develop new therapeutic agents and imaging diagnostics^29^.

Additionally, treatment with neutralizing monoclonal antibodies against GRP effectively inhibited the growth of oral cancer cells, demonstrating inhibitory effects both in vivo and in vitro^18,28^. The combination of a GRPR antagonist and EGFR inhibitor showed antitumoral effects with a high proportion of apoptotic cells in HNSCC, suggesting that targeting GRPR could enhance treatment efficacy^30^. Furthermore, GRPR expression in primary HNSCC tumors has been associated with lymph node metastasis, extracapsular infiltration, and tumor progression^28^.

Few studies have examined the relationship between GRPR expression and patient outcomes in head and neck and esophageal cancers^15,18^. Prior studies suggested a trend toward poorer survival with increasing GRPR levels, although statistical significance was not achieved, likely due to the limited number of patients with complete follow-up^18^. In our cohort, we observed a similar trend: high GRPR expression (> 50% stained cells) was associated with inferior survival. In contrast, Egloff^15^ and colleagues found no association between GRPR expression and disease-free survival in head and neck tumors.

Given the expression of GRPR across malignancies, numerous radiolabeled GRPR agonists and antagonists have been developed for imaging and therapy. In particular, several GRPR-targeting agents have advanced through preclinical and clinical evaluation, especially in prostate and breast cancers^31,32^. This experience supports extending GRPR-targeted diagnostics and therapeutics to other malignancies exhibiting GRPR overexpression, such as squamous cell carcinomas, warranting further investigation in this setting.

It is important to highlight that in our study we used a proprietary anti-GRPR antibody developed by Ziel Biosciences (Patent number BR 10 2014 007315-9). This antibody was previously tested and validated in a peer-reviewed study, demonstrating high sensitivity, specificity and accuracy for identifying GRPR in cervical precursor lesions and cancer^33^. Its performance supports the feasibility of GRPR immunohistochemistry for improving the detection of head and neck and esophageal SCC. Furthermore, it is important to acknowledge some limitations of our study. Our cohort was predominantly male, with an imbalance between tumor and normal tissues, and heterogeneity in anatomic site, stage and treatment, which may have introduced variability into the GRPR prognostic analyses. While these limitations do not diminish the relevance of our findings, future studies with larger, prospectively collected, and more homogeneous cohorts are needed to refine and confirm the prognostic value of GRPR.

## Conclusion

Our findings demonstrate the overexpression of GRPR in head and neck and esophageal SCC, with high GRPR levels correlating with worse survival. The anti-GRPR immunohistochemistry assay showed high sensitivity, specificity, and accuracy. These findings support GRPR as a candidate diagnostic and possible prognostic biomarker and warrant validation in larger, histologically and clinically homogeneous cohorts with standardized IHC scoring will be valuable to confirm the clinically meaningful effects.

## Data Availability

The data that support the findings of this study are available on request from the corresponding author. The data are not publicly available due to privacy or ethical restrictions.
